# The Effect of the Excisional Biopsy in the Detection of the Sentinel Lymph Node By Lymphoscintigraphy and Intraoperative Gamma Probe in Breast Cancer

**DOI:** 10.4274/MIRT.28

**Published:** 2011-12-01

**Authors:** Pelin Arıcan, İrfan Peksoy, Seniha Naldöken, Betül Bozkurt

**Affiliations:** 1 Ankara Numune Training and Research Hospital, Nuclear Medicine, Ankara, Turkey; 2 Ankara Numune Training and Research Hospital, General Surgery, Ankara, Turkey

**Keywords:** Breast cancer, sentinel lymph node, lymphoscintigraphy, intraoperative gamma probe

## Abstract

**Objective:** Sentinel lymph node (SLN) scintigraphy is used widespread in breast cancer, but the effect of the radionuclide agent, injection technique, the method of biopsy, tumor localization, breast size remain controversial. We examined the effects of the excisional biopsy in the rate of the SLN identification with lymphoscintigraphy (LS) and intraoperative gamma probe (IGP).

**Material and Methods:** One hundred patients (age range: 28-79 yr) with breast cancer were included in the study. They consisted of two groups: Group 1; there were 58 patients without excisional biopsy Group 2; there were 42 patients with excisional biopsy LS: 2 hours before the operation, 37 MBq/ ml Tc 99m colloidal rhenium sulphide was injected at the periaerolar region intradermally Anterior and lateral static images were acquired. IGP: The hot spot of greatest radioactivity were marked on the skin during the surgery with IGP and removed. Excised SLNs were examined with frozen section. After that histopathological and immunohistochemical examinations were performed.

**Results:** SLNs were found in all patients in group 1 (100%), in 39 patients of group 2 (93%) with LS. SLNs were excised in 57 of the 58 patients of group 1 (98%), in 38 of the 42 patients of group 2 (90%) with IGF. Metastases were found in SLNs in 27 patients (28%). Axillary dissection was performed in these patients.

**Conclusion:** According to results of our study the excisional biopsy was not the only factor but also other factors such as breast mass, calcified or metastatic lymph node may be affected the success rate of the SLN.

**Conflict of interest:**None declared.

## INTRODUCTION

Breast cancer is still a major health problem in women. Axillary lymph node status is the most important prognostic factor in patients with early breast cancer (1,2). Axillary lymph node metastasis is found approximately in 40% of these patients. In the remaining 60%, there is no therapeutic benefit from total axillary lymphadenectomy (3,4). However, the potential morbidity after axillary dissection is relatively high and may cause significant complications (5,6).

The sentinel lymph node (SLN) is the first lymph node to which the tumor is drained and it is the first to become involved in a metastasis from the tumor (7). SLN biopsy is the best procedure for the axillary lymph node status. The identification of the SLN plays an important role in surgical planning and in the management of breast cancer (7,8). There have been different techniques in the detection of the SLN mapping such as blue dye (9), radionuclide methods (lymphoscintigraphy and intraoperative gamma probe) (10,11,12,13,14) and simultaneous use of both. (12,13,14).

Although the SLN biopsy is used widespread; the best agent, injection technique, the method of biopsy, tumor localization and breast size remain controversial. Surgeon's experience is another factor in the determination of SLN. The previous excisional biopsy may affect the visualization of lymphatic drainage and SLN due to distruption of normal lymphatic pathways (15,16,17,18,19).

In our study, we investigated the effect of the excisional biopsy in the rate of the sentinel lymph node identification with lymphoscintigraphy and intraoperative gamma probe.

## MATERIALS AND METHODS

**Patient Population:** One hundred patients (age range: 28-79 yr; mean age: 53.5±25.5) with stage I-II breast cancer were included in the study. None of them had axillary palpable lymph nodes, multicentric tumor and history of prior radiotherapy. Informed consent was obtained from all patients. The characteristics of the patients are summarized in Table 1. The study consisted of two groups: Group 1; there were 58 patients without excisional biopsy. Group 2; there were 42 patients with excisional biopsy.

**Lymphoscintigraphy (LS):** On the day before the operation, Tc 99m colloidal rhenium sulphide (Nanocis, CIS) was injected at the periareolar region intradermally at four different locations in two groups. Total injection activity was 37 MBq/ml. After injection, each patient was instructed to massage the injection side for a few minutes. Anterior and lateral static images were obtained for 5 minutes using a single head gamma camera (Mediso or Elscint SPX) equipped with a low energy-high resolution collimator. The energy peak was set on 140 keV and 20% window. Counts were collected on a 256x256 matrix. If SLN was not visualized, delayed imaging was done at 2 hours after injection. However, delayed static images could not be taken from some patients because of the hurry of the operating room. Images were evaluated qualitatively by 2 nuclear medicine specialists.

**Intraoperative gamma probe (IGP):** Intraoperative localization of SLN was performed with IGP (Europrobe). All operations were performed by the same surgeon. The IGP was covered with a sterile endoscopic probe cover. The breast mass, axillary, supraclavicular, infraclavicular and internal mammary regions were investigated with IGP before the incision. The hot spot of greatest radioactivity was marked on the skin. The radioactive node(s) were removed and also counted ex vivo. Excised SLNs were sent to the pathologist for frozen section examination. If the frozen section was positive for metastases, total axillary dissection was performed.

**Pathological examination:** Histopathological examination was performed by microscopic examination with hematoxylin- eosin and immunohistochemical techniques.

**Statistical Analysis**

Mann-Whitney U was used to compare two groups.

## RESULTS

**The Results of LS and IGP**

**Figure 1:**
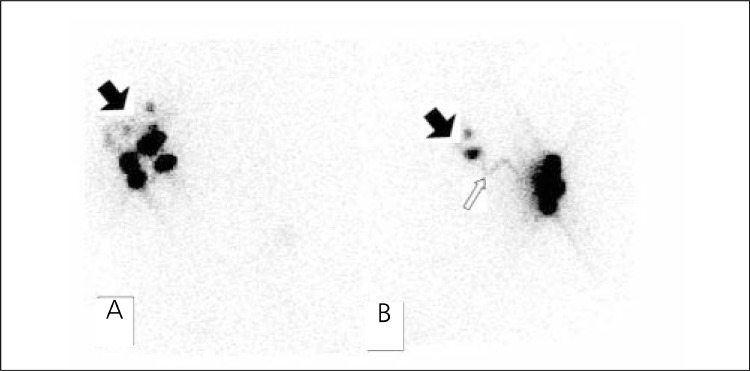
A patient without excisional biopsy. Anterior (A) and right lateral(B) static images show four SLNs (black arrow) and lymphatic pathways(white arrow)

**Group 1:** The results of LS and IGP in Group 1 are shown in Table 2. Lymphatic drainage through the axillary region and SLNs were found in all patients with LS. The detection rate was 100% (Figure 1). Only one patient showed internal mammarial drainage. SLNs were detected in 57 of the 58 patients with IGF! The detection rate was 98%. SLN was not detected with IGP in one patient (false negative). False positive results were not found with IGP.

**Figure 2:**
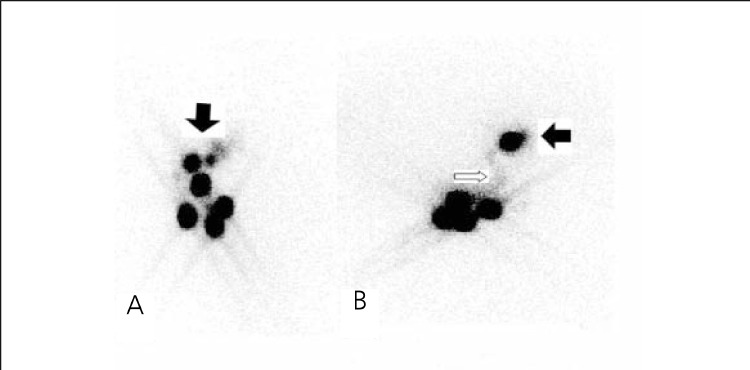
The patient with excisional biopsy. (A) There are three SLN inanterior image (black arrow) (B) lymphatic drainage pathway (white arrow)and SLN (black arrow) in left lateral image

**Group 2:** The results of LS and IGP in Group 2 are shown in Table 2. Lymphatic drainage through the axillary region and SLNs were found in 39 of 42 patients with LS. The detection rate was 93% (Figure 2). In 3 patients, no SLN was detected with LS. None of the patients showed extra-axillary drainage. SLNs were detected in 38 of the 42 patients with IGP The detection rate was 90%. In 4 patients, no SLN was detected with IGP (one patient false negative, 3 patients true negative). False positive results were not found with IGF!

There was no statistical difference between two groups (p>0.05).All SLNs (except 2 patients) which are found with LS and IGP were excised in both groups. In 5 of the 100 patients (one patient in group 1, four patients in group 2), SLNs were not detected with IGP Characteristics of these patients are presented in Table 3. In 3 of 5 patients, SLN and lymphatic drainage were not detected with both LS and IGP These patients were in Group 2. In 2 of 5 patients, SLNs and lymphatic drainage were found with LS, but they were not detected with IGP (one patient in group 1, one patient in group 2).

**Pathology Results:** Metastases were not found in excised SLNs in 68 of 95 patients by histochemical and immunohistochemical examination (72%). However, metastases were found in SLNs in 27 patients (28%). Axillary dissection was performed in these patients. Axillary metastasis was found in 15 patients (15%).

## DISCUSSION

Breast cancer is a major malignancy for women and the pathologic status of axillary lymph nodes are very important determinants of prognosis. Axillary node dissection is an invasive and complicated procedure for staging the axillary area and has no benefits for therapy. The pathologic examination of the SLN is essential to evaluate the status of the axillary region. If it is not metastatic, axillary lymphadenectomy may not be necessary.

LS, blue dye and IGP may be used separately or combined to detect lymphatic drainage and SLN(12,13,14). Although LS and IGP are increasingly used for detection of SLN, injection site, type of radioactive agent, volume, dose and timing are still controversy. In the literature, several authors have reported that previous biopsy had no effect on the success of the SLN identification (15,19). However some studies hypothesize that biopsy cavity may affect the SLN identification rate (15,16,17,18,19). The large excisional biopsy cavity may cause distruption of normal anatomic lymphatic pathways. Therefore, the detection rate of SLN may be reduced.

In our study; the comparison of Group 1 and Group 2 was not statistically different. The detection rate of LS and IGP in Group 1 and Group 2 were found 100%, 98%,95% and 90%, respectively. The identification rate of SLNs by using LS and IGP was compatible with the results of the literature (12,13,14,20,21).

Different injection techniques are used in the detection of SLN. Currently, periareolar intradermal injection techniques are preferred because of the rapid visualization and high rate of SLN detection, regardless of the localization of the tumor (18,21). The success rate of SLN detection is higher in superficial injections such as intradermal and subdermal than deeper injections such as peritumoral and intratumoral (22). On the other hand, the detection rate of internal mammarian drainage is higher with deeper injection than superficial injection (18,19,20,21,22). The periareolar injection can eliminate the effects of excisional biopsy, nonpalpabl tumor and multicentric tumor (23). Therefore, we preferred periareoler injection technique regardless of the type of biopsy. Other injection techniques such as peritumoral injection can affect identification of SLN, due to excisional cavity. In one patient, internal mammarian drainage was seen with LS in Group 1. Internal mammarian drainage did not change the surgery planning. The detection rate of the extraaxillary drainage was very low in our study. This situation can be explained that delayed static images could not be taken in some patients, because of the hurry of the operating room. These patients were sent to surgery earlier than usual. The success rate of extraaxillary lymphatic drainage with periareolar intradermal radioactivity injection is lower than other injection techniques (18,19,20,21,22).

Some SLNs were not detected with LS and IGP SLN activity may be suppressed by the injection site activity. Small size, calcification or full of metastatic cells in the lymph node may also prevent visibility. The SLN may not be detected with IGP when the operation time is too late (18,19,20,21,22). In our study, SLN and lymphatic drainage were not detected with both LS and IGP in 3 of 5 patients. These patients were in Group 2. SLNs and axillary metastasis were found by pathological examination in these patients. SLNs could not be detected with LS and IGP when lymph nodes are full of metastatic cells. In 2 of 5 patients, SLNs and lymphatic drainage were found with LS, but they were not detected with IGP (one patient in group 1, one patient in Group 2). We thought that, this status could be based on late operation time in these patients.

## CONCLUSION

SLN biopsy is the best procedure to determine the axillary lymph node status. LS and IGP have important roles for the detection of the SLN. There are many factors affecting the detection of SLN. One of these factors may be previous excisional biopsy. According to the results of our study, the excisional biopsy does not affect the success rate of the SLN, if radiopharmaceutical is injected at the periareolar region.

## Figures and Tables

**Table 1:**
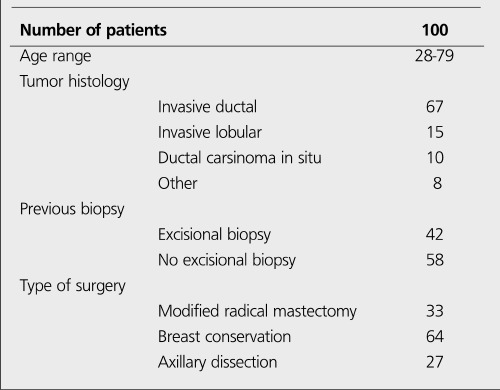
The characteristics of the patients

**Table 2:**
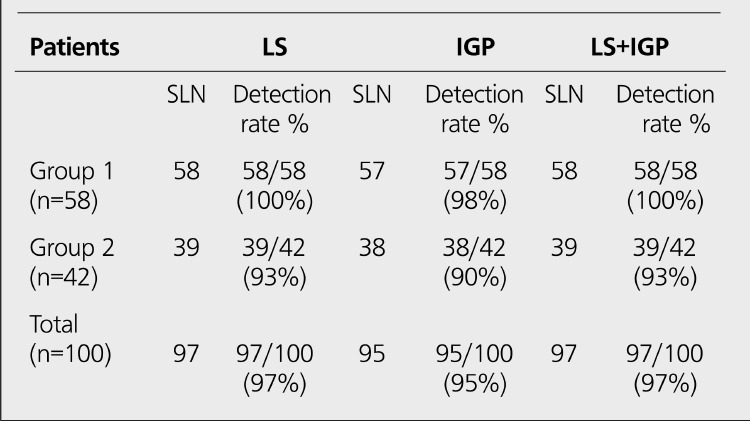
The results of SLN identification in Group 1 and Group 2

**Table 3:**
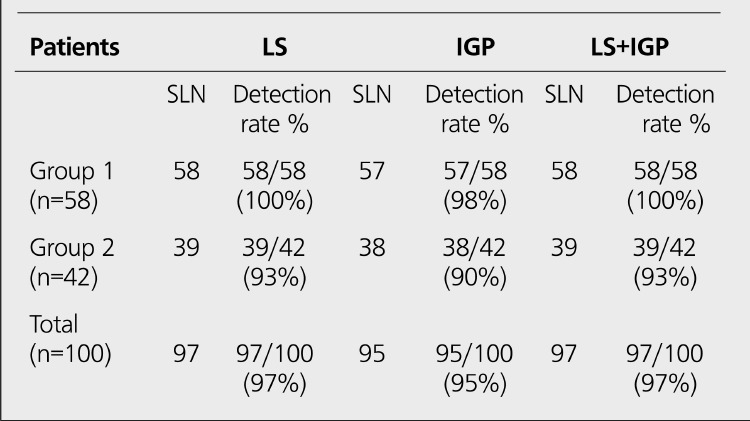
The results of the patients with no SLN detected with IGP
